# Carnosine ameliorates dexamethasone-induced muscle atrophy with associated modulation of ubiquitin ligases and oxidative stress in C57BL/6J female mice

**DOI:** 10.1016/j.crphys.2025.100169

**Published:** 2025-10-14

**Authors:** Md Mizanur Rahman, Anayt Ulla, Honomi Ogura, Haruka Tsuda, Takayuki Uchida, Tomoya Fukawa, Takeshi Nikawa

**Affiliations:** a Department of Nutritional Physiology, Institute of Biomedical Sciences, Tokushima University Graduate School, 3-18-15 Kuramoto-Cho, 770-8503, Tokushima, Japan; b Department of Urology, Institute of Biomedical Sciences, Tokushima University Graduate School, 3-18-15 Kuramoto-Cho, 770-8503, Tokushima, Japan

**Keywords:** Carnosine, Dexamethasone, Muscle atrophy, Oxidative stress, Ubiquitin ligase, C57BL/6J mice

## Abstract

Muscle atrophy, characterized by a decline in muscle mass and function, has limited treatment options, highlighting the need for further research. In this study, we investigated the effect of carnosine, a dipeptide with well-established antioxidant properties, on dexamethasone (Dex)-induced muscle atrophy in female C57BL/6J mice. Dex (10 mg/kg body weight) reduced muscle weight, cross-sectional area (CSA), and myosin heavy chain (MyHC) protein expression, while elevating the expression of the muscle atrophy–related ubiquitin ligases Atrogin-1 and Muscle RING-finger protein-1 (MuRF1). Dex also increased oxidative stress, leading to upregulation of the oxidative stress–sensitive ubiquitin ligase Cbl-b and downregulation of IRS-1. Notably, a 21-day treatment with carnosine (300 mg/kg body weight) significantly mitigated Dex-induced reductions in muscle mass, myofiber CSA, and MyHC protein, while suppressing ubiquitin ligase expression and preserving IRS-1 levels. Carnosine likewise decreased oxidative stress and the associated Cbl-b upregulation. These findings suggest that carnosine is a promising therapeutic candidate for managing Dex-induced muscle atrophy.

## Highlights

• **Carnosine prevented dexamethasone-induced muscle atrophy by improving myofiber CSA in mice.**
• **Carnosine effectively attenuated Dex-induced degradation of fast and slow MyHC proteins.**
• **Carnosine suppressed muscle-atrophy–associated ubiquitin ligases.**
• **Carnosine acts as a strong antioxidant and reduces oxidative stress.**

## 1 Introduction

Healthy muscles are essential for the physiological functions of the body. A decline in muscle mass and strength, known as muscle atrophy, can significantly impact the overall health and quality of life. Various physiological and pathological factors, such as ageing, cancer, diabetes, physical inactivity, nutritional deficiencies, and the use of glucocorticoid (GC) drugs, can cause muscle atrophy (Yin et al., 2021). GCs are among the most widely used clinical agents because of their anti-inflammatory and immunosuppressive properties. However, prolonged or high doses of this drug can lead to various side effects, including osteoporosis, muscle wasting, and weakness (Oray et al., 2016)**.** Dexamethasone (Dex), a potent and commonly used GC drug, has been shown to induce muscle atrophy both *in vivo* and *in vitro (*Massaccesi et al., 2016; Ulla et al., 2022*)*. Dex binds to the GC receptor, activates the ubiquitin-proteasome system (UPS), and enhances the expression of the muscle-specific ubiquitin E3 ligases MAFbx/Atrogin-1 and Muscle RING-finger protein-1 (MuRF1) (Schakman et al., 2013). Activation of UPS accelerates muscle protein degradation and impairs protein synthesis via E3 ubiquitin ligase activity.

Several *in vitro* and *in vivo* studies have shown that Dex-induced muscle atrophy is closely associated with oxidative stress in skeletal muscles (Ulla et al., 2022). The accumulation of reactive oxygen species (ROS) upregulates the ubiquitin ligase casitas B-lineage lymphoma proto-oncogene-b (Cbl-b) that ubiquitinates and degrades insulin receptor substrate-1 (IRS-1) (Nakao et al., 2009; Uchida et al., 2018). The insulin/IGF-1 pathway is a key regulator of muscle growth (Lin et al., 2021). Degradation of IRS-1 further impairs the insulin-like growth factor-1 (IGF-1) signalling pathway, activating FoxO3a, which induces the expression of the E3 ubiquitin ligase MAFbx/Atrogin-1 and MuRF-1(Sandri et al., 2004; Stitt et al., 2004). The impairment of the insulin/IGF-1 signalling pathway leads to the dephosphorylation of FoxO3a, which promotes the translocation of the FoxO3a from the cytoplasm to the nucleus and promotes muscle atrophy genes (O'Neill et al., 2016). Therefore, targeting oxidative stress is a promising approach for ameliorating Dex-mediated skeletal muscle atrophy.

Glucocorticoid-induced skeletal muscle atrophy is closely tied to activation of the ubiquitin–proteasome system (UPS). Key studies show that the E3 ligase MuRF1 is essential for glucocorticoid-induced muscle loss, as MuRF1-null mice are protected and display altered atrophy-related gene expression (Furlow et al., 2013; Baehr et al., 2011). Glucocorticoid receptor (GR) signaling cooperates with FOXO to drive MuRF1 expression (Waddell et al., 2008), and muscle-specific GR activity alone can trigger atrophy (Watson et al., 2012). These findings define a GR–FOXO–MuRF1 pathway as a central driver of muscle protein degradation. Because oxidative stress can amplify UPS activity, we investigated whether carnosine, a naturally occurring antioxidant, can attenuate dexamethasone-induced muscle atrophy and influence this pathway.

Peptides play a multifaceted role in muscle health, influencing growth, repair and recovery through various mechanisms(Hur et al., 2024; Lee et al., 2024). Dipeptides composed of two amino acids linked by the peptide bond play a significant role in muscle health by enhancing muscle protein synthesis, aiding recovery, improving nutrient absorption, regulating muscle atrophy and providing antioxidant benefits (Li et al., 2022; Yang et al., 2022). They may enhance protein synthesis by providing a quick source of amino acids readily absorbed and utilized by the body. Moreover, they can aid muscle recovery by promoting the repair of muscle fiber damage during exercise helping to reduce muscle soreness and inflammation(Jimi et al., 2021). Carnosine (β-alanin-L-histidine) is a natural dipeptide synthesized in human tissues, with high concentrations in muscles, heart, and brain tissues (Jukić et al., 2021). Its functions are particularly relevant to muscle health as an antioxidative and to maintain proteostasis (Agrawal et al., 2022). Carnosine acts as a buffer against acid accumulation during high-intensity exercise (Varanoske et al., 2017). Biologically, carnosine exerts free radical scavenging, physiological pH buffering, anti-glycation, and anti-lipid peroxidation activities(Alexander et al., 1999; Hipkiss et al., 2001). Moreover, it is a cardioprotective, anti-inflammatory, anti-obesity, anti-diabetic, anti-ageing, and neuroprotective agent (Hariharan et al., 2024; Maugeri et al., 2023; Schaalan et al., 2018). Previously, we reported the effect of carnosine on C2C12 myotubes (Rahman et al., 2024). However, its *in-vivo* effect on muscle atrophy has not yet been shown. Therefore, this study aimed to investigate the potential protective effects of carnosine against dexamethasone-induced skeletal muscle atrophy in mice, with particular attention to markers of protein degradation and oxidative stress.

## 2 Materials and methods

### 2.1 Reagents and chemicals

Carnosine was graciously gifted by Hamari Chemicals Ltd. (Osaka, Japan). Dexamethasone was obtained from Sigma-Aldrich (St. Louis, MO) and Isogen™ from Nippon Gene (Tokyo, Japan). All other chemicals and reagents used were of analytical grade.

### 2.2 Experimental design

Female C57BL/6j mice (12–13 weeks old, weighing 20–22 g) were purchased from Japan SLC (Shizuoka, Japan) and maintained under standard conditions: temperature (24 ± 1 °C), humidity (55 ± 10 %), and a 12-h dark/light cycle. The mice were given a standard solid food and water diet throughout the experimental period. After 1 week of acclimatization, the mice were divided into four experimental groups (n = 6/group): control (vehicle), Dex (10 mg/kg BW), carnosine (300 mg/kg BW), and Dex + carnosine. Carnosine was dissolved in water and administered orally for 21 days. Dex was dissolved in saline and intraperitoneally injected during the last 10 days of the experimental period. The control and Dex groups received water (vehicle for carnosine) at the same volume as that of carnosine for 21 days. Moreover, the control mice were injected with saline (vehicle for Dex) for 10 days. The body weight of the mice was recorded daily throughout the experiment. After 21 days of treatment, the mice were fasted for 6 h and then euthanized. Then, the blood samples and tissues from muscles, including gastrocnemius (GA), tibialis anterior (TA), soleus (SOL), and extensor digitorum longus (EDL), were collected. The blood samples were centrifuged at 5870 g for 15 min at 4 °C to separate the plasma. The harvested muscle tissues were weighed. Both samples were stored at −80 °C for further investigation.

### 2.3 Cross-sectional area (CSA) analysis

After euthanasia, gastrocnemius (GA) muscles were rapidly excised and frozen on a staining wood block in isopentane chilled with liquid nitrogen. Frozen muscles were sectioned at 10 μm thickness using a cryostat and stained with hematoxylin (1 min) and eosin (3 min) at room temperature.

Muscle histology was imaged with a phase-contrast microscope (BIOREVO BZ-9000; Keyence, Osaka, Japan) and analyzed using BZ-II Analyzer software (Keyence). For CSA quantification, 10 randomly selected fields per muscle were captured at 20 × magnification in a blinded manner. Each field contained approximately 150–250 fibers (fiber area range 500–4000 μm^2^), yielding a total of about 1500–2500 fibers per muscle. Images were collected from multiple regions of each GA muscle to ensure representative sampling.

### 2.4 Protein analysis

For immunoblotting, the GA muscle tissues were homogenized in lysis buffer (10 vol) containing 50 mM Tris HCl (pH 7.5), 150 mM NaCl, 5 mM EDTA, 10 mM NaF, 2 mM Na3VO4, 1 % Triton X-100, cOmplete™ Protease Inhibitor Cocktail, (Roche Diagnostics, Cat. No. Cat. No. 11697498001), and 10 μM MG-132. The homogenate was then centrifuged at 12,000 g for 15 min at 4 °C. The protein concentration was measured using the Pierce™ BCA Protein Assay Kit (Thermo Fisher Scientific), with bovine serum albumin as the standard, following the manufacturer's instructions. Then, 20 μg of protein per sample was loaded onto an 8 % sodium dodecyl sulfate-polyacrylamide gel, electrophoresed at 300 V, and transferred to polyvinylidene difluoride membranes. The membranes were blocked for 1 h in 4 % block ACE™ (DS Pharma Biomedical) dissolved in Milli-Q water. Next, the membranes were incubated with the primary antibodies overnight at 4 °C, washed, and further incubated for 1 h at room temperature with the secondary antibodies. Blots were analyzed using a C-DiGit scanner (LI-COR Biosciences). The primary antibodies included, total FoxO3a and phosphorylated FoxO3a (Ser^253^) (1:1000) (Invitrogen), Atrogin-1 and MuRF-1 (1:5000) (abcam), and 4-hydroxy-2-nonenal (HNE) (1:5000), IgG mouse and rabbit secondary antibodies (1:5000) (Cell signaling, MA, USA) were used.

Western blotting for the total, fast (Sigma-Aldrich), and slow (Novus Biologicals, Littleton, USA) types of MyHC (1:200), IRS-1 (1:50), Cbl-b (1:200) (Cell signaling, MA, USA), IgG mouse and rabbit secondary antibodies (1:100) (Cell signaling) was performed using the ProteinSimple™ WES system. For this, 0.5 μg of protein was mixed with the Simple Western sample buffer and 5X Fluorescent Master mix and denatured at 95 °C for 5 min. Then, the samples were loaded into the designated wells of a WES microplate, along with the primary and secondary antibodies and the chemiluminescent substrate, according to the manufacturer's instructions. The microplate and the appropriate capillary cartridge were placed into the system for automated western blotting. After approximately 3 h, the software-generated results were analyzed to assess the protein expression levels.

### 2.5 Quantitative real-time polymerase chain reaction

RNA was extracted from the GA muscle homogenates using the ISOGEN™ reagent (Nippon Gene, Tokyo, Japan), and its concentration was determined using a Nanodrop 1000 spectrophotometer (Thermo Fisher Scientific). Subsequently, 1 μg of RNA was reverse transcribed into cDNA. Real-time reverse transcription polymerase chain reaction (RT-PCR) was conducted using the SYBR Green dye and the Step One Plus™ Real-Time PCR system (Applied Biosystem, CA, USA), with the Brilliant III Ultra-Fast SYBR® Green QPCR Master Mix™ (Agilent, Texas, USA). The 18S ribosomal RNA was used as the internal control for normalization. The PCR primer sequences are provided in Table 1.

**Table 1:** Primers used for polymerase chain reaction.

Target gene		Sequence	Length (bp)
MAFbx1/atrogin-1	S	GGCGGACGGCTGGAA	101
	AS	CAGATTCTCCTTACTGTATACCTCCTTGT	
MuRF-1	S	TGTCTGGAGGTCGTTTCCG	183
	AS	CTCGTCTTCGTGTTCCTTGC	
Cbl-b	S	GAGCCTCGCAGGACTATGAC	222
	AS	CTGGCCACTTCCACGTTATT	
SOD1	S	ACCAGTGCAGGACCTCATTTTAA	78
	AS	TCTCCAACATGCCTCTCTTCATC	
Catalase	S	ATGGCTTTTGACCCAAGCAA	69
	AS	CGGCCCTGAAGCTTTTTGT	
18Sr	S	CATTCGAACGTCTGCCCTA	119
	AS	CCTGCTGCCTTCCTTGGA	

18Sr, 18S ribosomal RNA; Cbl-b, Casitas B-lineage lymphoma proto-oncogene-b; MAFbx1, muscle atrophy F-box protein 1; MuRF-1, muscle ring finger protein-1; SOD1, superoxide dismutase.

### 2.6 Analysis of the oxidative stress parameters

The GA muscle tissues were homogenized in lysis buffer and centrifuged at 13,200 g for 15 min at 4 °C. The resulting supernatants were collected to evaluate oxidative stress parameters, including malondialdehyde (MDA) and advanced oxidation protein products (AOPP), following previously described methods (Rahman et al., 2017). MDA, a lipid peroxidation by-product and an oxidative stress marker in plasma and muscle tissues, was measured colorimetrically using the thiobarbituric acid reactive substance assay as described previously (Ulla et al., 2017). The AOPP levels, another key indicator of oxidative stress, in plasma and muscle tissue samples were evaluated using a colorimetric method outlined by Rahman et al. (2017). The AOPP concentrations were expressed as nmol/mL chloramine-T equivalents, determined using a chloramine-T standard curve. The absorbance of the reaction mixture, which contained plasma or tissue diluted in phosphate buffer solution, potassium iodide, and acetic acid, was measured immediately at 340 nm against a blank. This method showed linearity in the 0−100 nmol/mL range at the specified wavelength.

### 2.7 Statistical analysis

Data are presented as mean ± standard error of the mean (SEM). Statistical analyses were performed using GraphPad Prism version 9.3.1 (GraphPad Software, San Diego, CA, USA). Group differences were evaluated by two-way analysis of variance (ANOVA) with Tukey's post-hoc multiple-comparison test. A p < 0.05 was considered statistically significant.

## 3 Results

### 3.1 The effect of carnosine on body weight

**Fig. 1:**
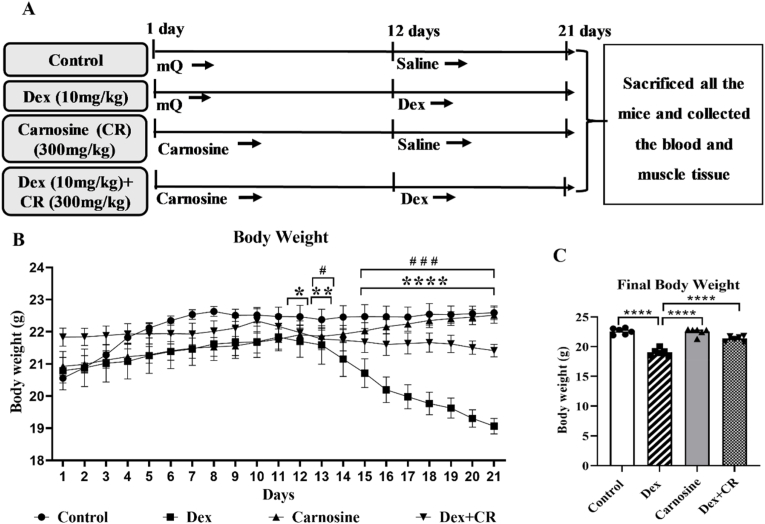
Carnosine attenuates dexamethasone-induced muscle atrophy in mice. (A) Schematic of experimental design. Mice were treated with carnosine (300 mg/kg body weight) orally for 21 days, dissolved in water. Dex (10 mg/kg body weight) was administered intraperitoneally, dissolved in saline, for the last 10 days only. (B) Body weight of the mice was measured daily throughout the experiment. (C) Final body weight of the mice after treatment. Results are presented as means ± SEM (standard error of the mean), (n = 6 per group). *∗P < 0.05* indicates a significant difference between the control and Dex-treated groups, while *#P < 0.05* indicates a significant difference between the Dex and Dex + CR groups*. ∗∗∗∗P < 0.0001.* Abbreviations: BW, body weight; Dex, dexamethasone; Dex + CR, dexamethasone with carnosine.

The body weights of the animals treated with carnosine and/or dexamethasone were monitored over the 21-day experimental period. The body weights of the Dex-treated mice reduced noticeably starting on day 13, with the difference being most significant between days 15–21 compared to the control mice (Fig. 1B). Co-treatment with carnosine (300 mg/kg body weight) alongside Dex significantly restored the body weight compared with the Dex-only group (Fig. 1B–C). In contrast, the carnosine-only treatment group showed no significant difference in body weight compared with the control mice.

### 3.2 The effect of carnosine on muscle weight

**Fig. 2:**
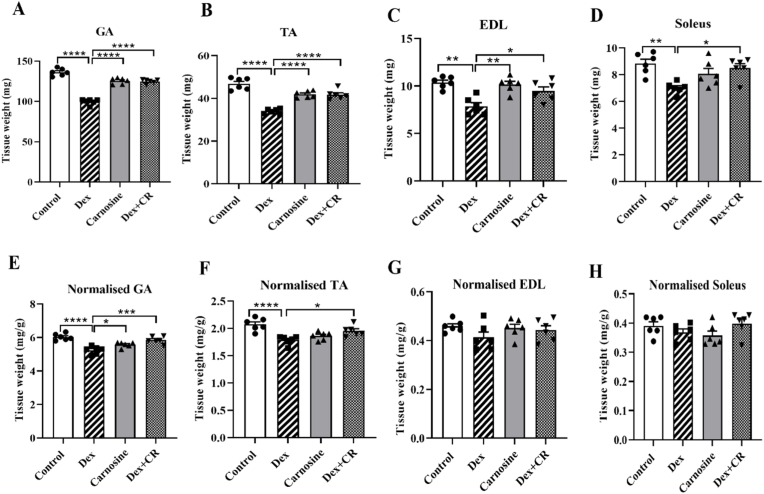
Carnosine attenuates dexamethasone-induced muscle atrophy and recovered muscle weight loss in mice. (A) Muscle weights of the gastrocnemius (GA). (B) Muscle weights of the tibialis anterior (TA). (C) Muscle weights of the extensor digitorum longus (EDL). (D) Muscle weights of the soleus (SOL). (E) Normalized weight of the GA muscle. (F) Normalized weight of the TA muscle. (G) Normalized weight of the EDL muscle. (H) Normalized weight of the SOL muscle. Mice were treated with carnosine (300 mg/kg body weight) orally for 21 days, dissolved in water. Dex (10 mg/kg body weight) was administered intraperitoneally, dissolved in saline, for the last 10 days only. Results are presented as means ± SEM (standard error of the mean), (n = 6 per group). *∗P < 0.05, ∗∗P < 0.01, ∗∗∗P < 0.001, ∗∗∗∗P < 0.0001.* Abbreviations: BW, body weight; Dex, dexamethasone; Dex + CR, dexamethasone with carnosine.

Various muscles in mice were weighed, and their total and normalized weights (muscle weight/body weight) were used as critical parameters for assessing muscle atrophy. In the Dex-treated mice, the total weight of the GA, TA, EDL, and SOL muscles was significantly reduced compared with the control group (Fig. 2A–D). Consistently, the normalized weights of the GA and TA muscles decreased, with no significant change in EDL and SOL muscles compared with the Dex treatment (Fig. 2E–H). Carnosine co-treatment with Dex significantly improved the weights of the GA, TA, EDL and SOL muscles compared with the Dex-only group (Fig. 2A–D). For normalized muscle weights, only the GA and TA muscles showed significant recovery with carnosine treatment, whereas the EDL and SOL normalized weights remained unaffected (Fig. 2E–H). In the carnosine-only group, no significant changes in muscle weights were observed compared with the control group (Fig. 2A–H).

### 3.3 The effect of carnosine on the CSA of myofibers

**Fig. 3:**
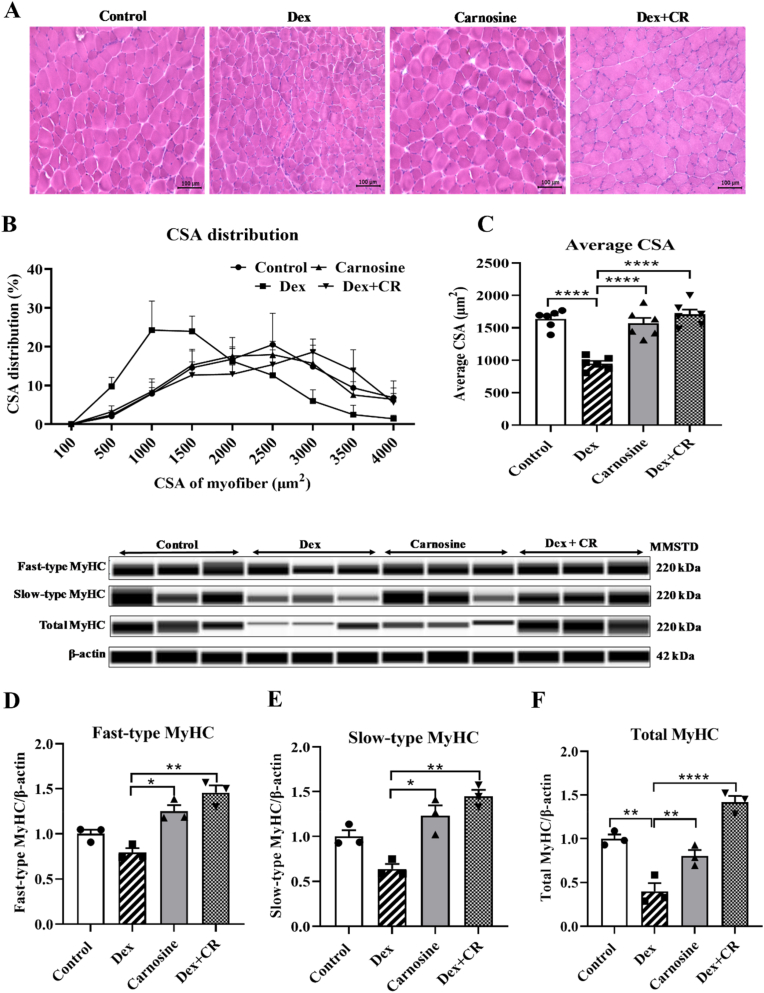
Carnosine attenuates dexamethasone-induced muscle atrophy with increased muscle cross sectional area (CSA) and muscle protein expression in mice. (A) H&E staining of GA muscle, (B) distribution of CSA in GA muscle, (C) average CSA in GA muscle. Protein expression by simple Western blot (D) Fast-type MyHC, (E) Slow-type MyHC and (F) Total MyHC. Mice were treated with carnosine (300 mg/kg body weight) orally for 21 days, dissolved in water. Dex (10 mg/kg body weight) was administered intraperitoneally, dissolved in saline, for the last 10 days only. Results are presented as means ± SEM (standard error of the mean), (n = 3 per group). *∗P < 0.05, ∗∗P < 0.01, ∗∗∗P < 0.001, ∗∗∗∗P < 0.0001.* Abbreviations: MMSTD, molecular mass standard; Dex, dexamethasone; Dex + CR, dexamethasone with carnosine.

To evaluate the impact of carnosine on the muscle CSA in Dex-treated mice, the GA muscle sections were stained with hematoxylin and eosin (Fig. 3A). The distribution of CSA and the average CSA were then analyzed (Fig. 3B and C). Dex treatment significantly reduced muscle CSA compared with the control group. Interestingly, Dex treatment induced a notable reduction in myofiber CSA in the Dex-alone group (Fig. 3B and C), which was significantly attenuated by carnosine co-treatment. In the Dex-only group, the CSA distribution predominantly ranged from 500 to 1500 μm^2^, with a dramatic reduction in larger muscle fibers (1500–4000 μm^2^) (Fig. 3B). Conversely, the percentage of larger fibers in the carnosine-treated mice was significantly higher than that in the Dex-only group (Fig. 3B).

### 3.4 The effect of carnosine on MyHC muscle protein expression

Dex treatment significantly reduced the levels of total MyHC proteins (*p ≤ 0.01*) in the GA muscle, although the decrease in the fast-type and slow-type MyHC levels was insignificant (Fig. 3D–F). Notably, carnosine treatment effectively mitigated the Dex-induced reduction of fast-type, slow-type, and total MyHC proteins compared with the Dex-only groups (Fig. 3D–F). In contrast, no significant adverse changes were observed in the levels of these proteins in the carnosine-treated group compared with all other experimental groups (Fig. 3D–F). These results suggest that Dex causes a reduction in the overall MyHC protein levels, which was ameliorated by carnosine.

**Fig. 4:**
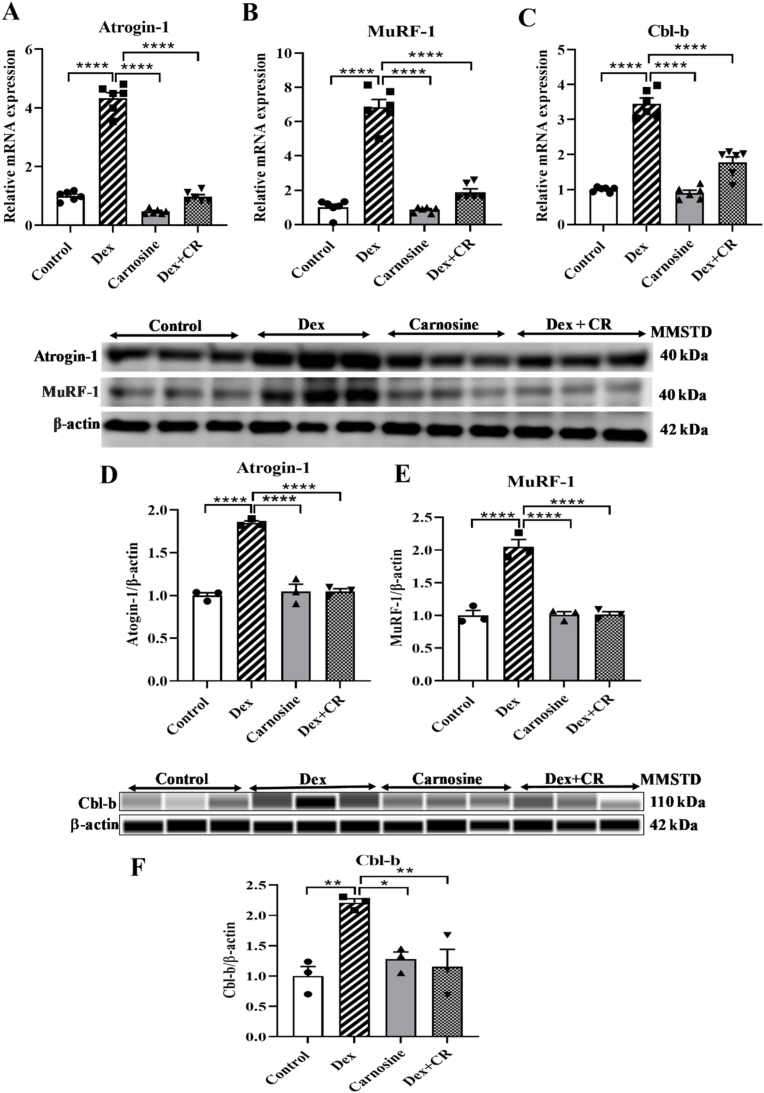
Carnosine attenuates dexamethasone-induced muscle atrophy by suppressing the mRNA expression and protein expression of ubiquitin ligases in mice. mRNA expression of (A) Atrogin-1, (B) MuRF-1 and (C) Cbl-b (n = 6 per group). Protein expression of (D) Atrogin-1, (E) MuRF-1 and (F) Cbl-b by western blotting (n = 3 per group). Mice were treated with carnosine (300 mg/kg body weight) orally for 21 days, dissolved in water. Dex (10 mg/kg body weight) was administered intraperitoneally, dissolved in saline, for the last 10 days only. Results are presented as means ± SEM (standard error of the mean), *∗P < 0.05, ∗∗P < 0.01, ∗∗∗P < 0.001, ∗∗∗∗P < 0.0001*. Abbreviations: MMSTD, molecular mass standard; Dex, dexamethasone; Dex + CR, dexamethasone with carnosine.

### 3.5 The effect of carnosine on the mRNA and protein expression of E3 ubiquitin ligases

Real-time-PCR was used to assess the effects of carnosine on the mRNA expression of the ubiquitin ligases Atrogin-1, MuRF-1, and Cbl-b in the GA muscles of Dex-treated mice (Fig. 4A–C). The protein levels of Atrogin-1, MuRF-1 and Cbl-b were measured in the mice with Dex-induced muscle atrophy using Western blotting (Fig. 4D–F). The mRNA expression of Atrogin-1, MuRF-1, and Cbl-b was significantly higher in the Dex-treated group than in the control group (Fig. 4A–C). In contrast, in the Dex + carnosine-treated group, the mRNA expression of these genes was significantly reduced compared with the Dex-only group (Fig. 4A–C). However, no significant changes in the mRNA expression of these genes were observed in the carnosine-only treated group (Fig. 4A–C).

The changes in the protein expression of Atrogin-1, MuRF-1 and Cbl-b were consistent with their mRNA levels (Fig. 4D–F). Dex treatment significantly increased the protein levels of Atrogin-1, MuRF-1 and Cbl-b compared with the control mice (Fig. 4D–F), and these elevated levels were effectively reduced by carnosine treatment (Fig. 4D–F). The carnosine-only treatment showed no significant changes in protein levels compared with the control group (Fig. 4D–F).

### 3.6 The effect of carnosine on IRS-1 and FoxO transcription factor

**Fig. 5:**
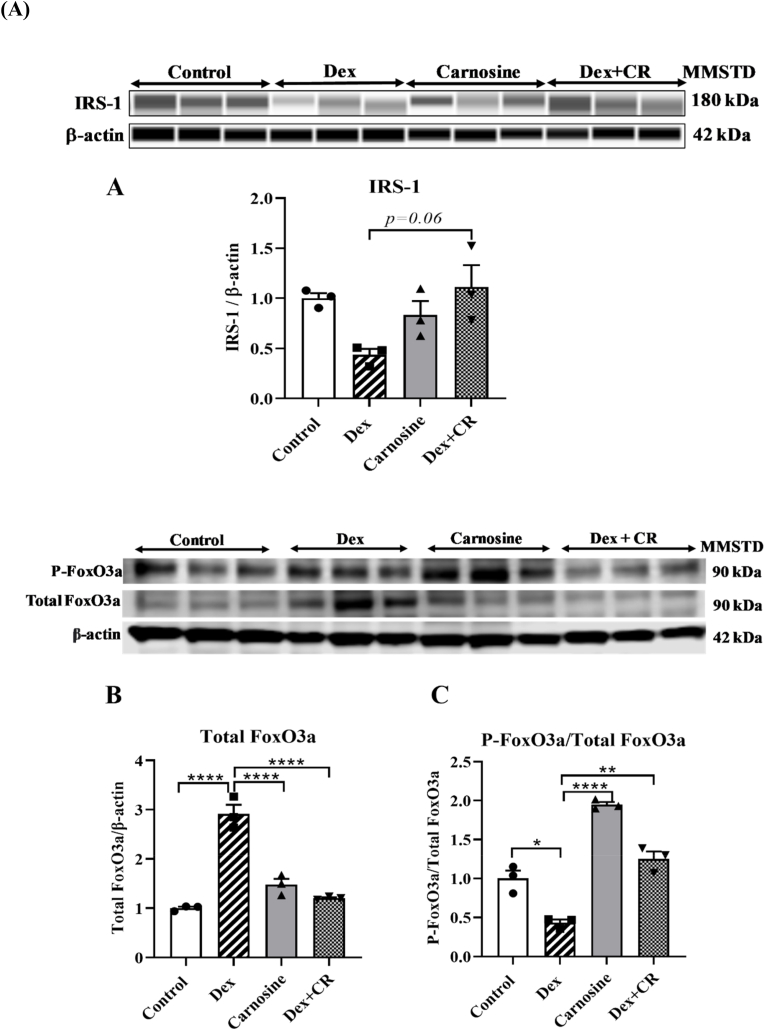
Carnosine attenuates dexamethasone-induced muscle atrophy with enhancing IRS-1 expression and suppressing FoxO3a activation in mice. Protein expression of (A) IRS-1 (B) Total Foxo3a and (C) P-Foxo3a/Total Foxo3a. Mice were treated with carnosine (300 mg/kg body weight) orally for 21 days, dissolved in water. Dex (10 mg/kg body weight) was administered intraperitoneally, dissolved in saline, for the last 10 days only. Results are presented as means ± SEM (standard error of the mean), (n = 3 per group). *∗P < 0.05, ∗∗P < 0.01, ∗∗∗P < 0.001, ∗∗∗∗P < 0.0001.* Abbreviations: MMSTD, molecular mass standard; Dex, dexamethasone; Dex + CR, dexamethasone with carnosine.

Western blot analysis revealed that dexamethasone treatment markedly reduced IRS-1 protein levels in gastrocnemius muscle compared with controls (Fig. 5A). Carnosine treatment alone showed no significant change relative to control. Notably, carnosine co-treatment tended to attenuate the Dex-induced reduction, with IRS-1 expression approaching control values (Dex + Carn vs Dex, p = 0.06), although this difference did not reach statistical significance.

Considering that FoxO3a is an upstream regulator of Atrogin-1 and MuRF-1, the effects of carnosine on the protein expression of total FoxO3a and phosphorylated FoxO3a (P-FoxO3a) were assessed (Fig. 5B–C). The total FoxO3a levels were significantly higher in the Dex-treated group than the control group (Fig. 5B). Interestingly, carnosine treatment significantly reduced the expression of total FoxO3a compared with the Dex-only group (Fig. 5B). Additionally, Dex treatment induced the dephosphorylation of FoxO3a compared with the control group (Fig. 5C). This effect was significantly reversed by carnosine, as shown by the higher levels of P-FoxO3a in the carnosine + Dex group than in the Dex group (Fig. 5C). Carnosine alone did not exert any significant changes and showed similar expressions as the control group (Fig. 5B–C).

### 3.7 The effect of carnosine on lipid peroxidation and oxidative stress

**Fig. 6:**
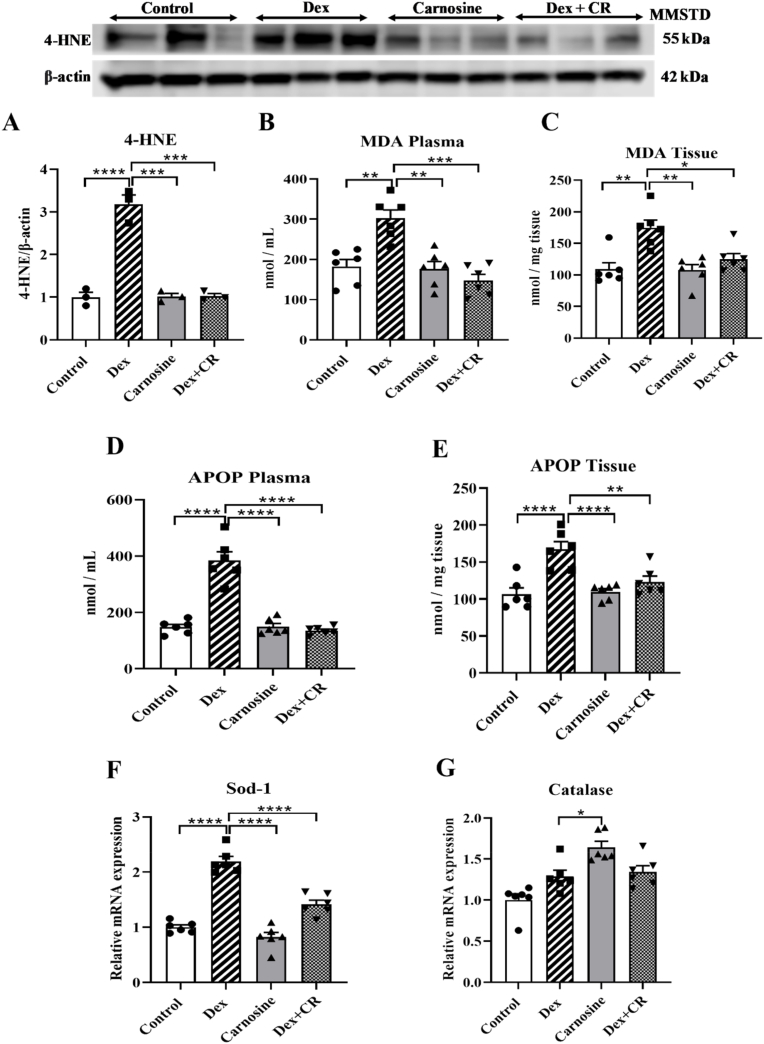
Carnosine attenuates dexamethasone-induced muscle atrophy with suppressing the lipid peroxidation and oxidative stress parameters in mice. (A) Protein expression of 4-HNE. (B–E) oxidative stress markers MDA and APOP in plasma and muscle tissue. (F) mRNA expression of antioxidant enzymes of Sod-1 and (G) Catalase. Mice were treated with carnosine (300 mg/kg body weight) orally for 21 days, dissolved in water. Dex (10 mg/kg body weight) was administered intraperitoneally, dissolved in saline, for the last 10 days only. Results are presented as means ± SEM (standard error of the mean), (n = 6 per group). *∗P < 0.05, ∗∗P < 0.01, ∗∗∗P < 0.001, ∗∗∗∗P < 0.0001*. Abbreviations: MMSTD, molecular mass standard; Dex, dexamethasone; Dex + CR, dexamethasone with carnosine.

Lipid peroxidation and ROS exacerbate protein breakdown, muscle damage, and muscle atrophy, making them potential targets for intervention. In this study, we measured the end products of lipid peroxidation; 4-hydroxynonenal (4-HNE) and MDA along protein oxidation marker AOPP, and the levels of antioxidant enzymes, including superoxide dismutase (SOD)-1 and catalase in Dex-induced mice. Dex treatment significantly increased the protein expression of 4-HNE compared with the control mice, which was effectively suppressed by carnosine treatment (Fig. 6A). Similarly, Dex treatment also significantly increased the MDA and AOPP levels in both plasma and tissue samples compared with the control group (Fig. 6B–E), and this increase was also effectively attenuated by carnosine treatment (Fig. 6A–E). The levels of 4-HNE, MDA, and AOPP in both the plasma and tissue samples of the carnosine-treated group were like those of the control group (Fig. 6A–E).

The mRNA levels of Sod-1 and catalase were higher in the Dex-treated mice compared with the control group (Fig. 6F and G), while those in the Dex + carnosine-treated group were lower compared with the Dex-treated group (Fig. 6F and G). In the carnosine-only group, there were no significant differences in the mRNA expression of these enzymes compared with the control group (Fig. 6F and G).

## 4 Discussion

This study investigated the potential anti-atrophic effects of carnosine, a dipeptide with potent antioxidant properties, against dexamethasone (Dex)-induced skeletal muscle atrophy in mice. Carnosine administration for 21 consecutive days significantly attenuated Dex-induced losses in body and muscle weight. It also reduced the Dex-mediated decrease in myofiber cross-sectional area (CSA) and prevented degradation of myosin heavy chain (MyHC) protein. In addition, carnosine suppressed the Dex-induced up-regulation of the ubiquitin ligases Atrogin-1, MuRF-1, and Cbl-b, while preserving IRS-1 expression, key molecular events associated with muscle atrophy. These findings are consistent with our previous *in vitro* study demonstrating carnosine's protective effects against Dex-induced muscle atrophy in C2C12 myotubes.

Several previous reports, including ours, have shown that Dex treatment elevated oxidative stress levels *in vitro* and *in vivo* (Kim et al., 2021; Oh et al., 2021; Ulla et al., 2021). Oxidative stress is closely associated with muscle atrophy (Powers et al., 2011; Seo et al., 2022; Lian et al., 2022). Increased oxidative stress causes the upregulation of Cbl-b ubiquitin ligase, which results in the ubiquitination and subsequent degradation of insulin receptor substrate-1 (IRS-1) (Uchida et al., 2018; Nakao et al., 2009). This further disturbs the IGF-1 signaling pathway, leading to the activation of FoxO3a, which stimulates the level of Atrogin-1 and MuRF-1, causing muscle protein degradation (Chen et al., 2022). In our study, carnosine significantly suppressed the Dex-induced ROS accumulation and oxidative stress, as evidenced by the reduced concentration of oxidative stress markers such as MDA, 4-HNE and AOPP. Reducing oxidative stress resulted in diminished Cbl-b and its downstream factors, such as FoxO3a. Dex significantly dephosphorylated the FoxO3a, which was noticeably reversed by the carnosine treatment, preventing FoxO3a entry into the nucleus, hence preventing the transcription of Atrogin-1 and MuRF-1. Furthermore, carnosine treatment effectively diminished the Dex-induced reduction in myofiber CSA. By downregulating ubiquitin ligases and oxidative stress markers, carnosine demonstrated a robust protective effect on muscle integrity, maintaining both MyHC protein levels and the myofiber CSA.

Carnosine has been identified as an effective anti-oxidative agent for muscle health due to its anti-inflammatory and antioxidant properties (Derave et al., 2010; Suzuki et al., 2002). It can attenuate muscle fatigue and improve mitochondrial function, thus enhancing athletic capabilities (Varanoske et al., 2017). Moreover, it acts as a pH buffer in muscle tissue, helping to maintain acid-base balance during high-intensity exercise (Sale et al., 2013). Furthermore, carnosine supplementation can regulate the contractile function of skeletal muscles (Dutka et al., 2012). Similarly, Dawson et al. reported that carnosine protects against ROS produced during exercise (Matthews et al., 2019). The level of carnosine is found to decline with ageing, therefore supplementation of it may help to mitigate the effect of sarcopenia (Wang et al., 2024). Consistent with these findings, our results provide further additional evidence indicating the potential effects of carnosine on muscle function, especially on muscle atrophy.

Dexamethasone has a significant effect on muscle tissue, particularly on myosin-heavy chain (MyHC) proteins which are the crucial component of muscle contractile apparatus. It can shift the muscle fiber type composition often promoting a transition from fast-twitch (Type-II) fibers to slow-twitch (Type-I) fibers that negatively impact on muscle performance and endurance (Fappi et al., 2019). In the current study, Dex decreased muscle weight especially GA and TA, along with attenuation of myofiber CSA and MyHC protein expression. Intriguingly, carnosine treatment ameliorated the reduction of muscle mass and myofiber CSA, accompanied by preservation of muscle contractile protein MyHC (Fig. 2, Fig. 3) and a reduction in the expression of the E3 ubiquitin ligases MuRF1 and MAFbx.

A significant advantage of carnosine is its safety profile (Bae et al., 2013). Although limited evidence suggests that carnosine might be toxic at normal dietary levels, most studies indicate that it does not exhibit any significant toxicity (Bae et al., 2013). A previous report found no side effects or toxicity in mice, even at the effective dose of 300 mg/kg body weight for 10 weeks of administration (Yılmaz et al., 2017). Furthermore, intravenous injection of carnosine at 2000 mg/kg body weight did not exhibit significant toxicity in rats (Bae et al., 2013). Subsequently, in a human clinical trial, the daily administration of 500 mg of orodispersible l-carnosine for six months was shown to improve physical performance and quality of life without adverse effects. (Lombardi et al., 2015). Therefore, carnosine can be regarded as safe for most individuals when consumed in moderate amounts from natural food sources such as meat and fish. Although it is widely recognized as a dietary component, carnosine does not yet have an official FDA “Generally Recognized as Safe” (GRAS) designation. When used as a dietary supplement, however, it is typically well tolerated (Solis et al., 2015). The overall safety profile and evidence for its ability to help preserve muscle mass and function support carnosine as a promising therapeutic candidate.

Our data show that carnosine supplementation blunts dexamethasone-induced muscle loss, reduces oxidative stress markers, and decreases expression of MuRF1, MAFbx, and Cbl-b. These findings align with and extend previous work identifying the GR–FOXO–MuRF1 signaling pathway as a core mediator of glucocorticoid-driven atrophy. Specifically, deletion of MuRF1 protects against muscle wasting caused by synthetic glucocorticoids and alters the atrophy-associated transcriptome (Furlow et al., 2013; Baehr et al., 2011). Moreover, GR and FOXO act synergistically to activate MuRF1 transcription (Waddell et al., 2008), and muscle-specific GR activation alone is sufficient to cause atrophy (Watson et al., 2012). By lowering oxidative stress and suppressing MuRF1 expression, carnosine may attenuate this established pathway, thereby preserving muscle mass. Although we did not directly measure GR signaling, the concordance between our observations and these seminal studies suggests that antioxidant modulation of the GR–FOXO–MuRF1 axis is a plausible mechanism for carnosine's protective effects, warranting further mechanistic investigation.

While our findings demonstrate that carnosine attenuates dexamethasone-induced skeletal-muscle atrophy, several limitations should be acknowledged. First, although we observed reduced oxidative-stress markers and down-regulation of MuRF1, MAFbx, and Cbl-b, we did not directly measure protein synthesis, total ubiquitination, proteasome activity, or IRS-1 ubiquitination; therefore, for precise mechanism of action further studies are warranted. Second, we did not monitor individual food intake or energy expenditure, which could influence body-weight changes. Finally, the duration of carnosine treatment was limited to a short-term preventive model, and long-term efficacy, pharmacokinetics, and potential off-target effects were not evaluated. These factors should be addressed in future studies to strengthen the translational relevance of our findings.

In conclusion, this study provides evidence that carnosine can ameliorate the side effects of glucocorticoid treatment. These findings can facilitate further research on carnosine as a potential intervention for several muscle-wasting conditions, such as cachexia, sarcopenia, and muscular dystrophy, in addition to Dex-induced muscle atrophy. However, additional studies are required to confirm the applicability of carnosine in human subjects. Furthermore, the long-term effects and pharmacokinetics of carnosine treatment should be explored.

## Author contribution

Md Mizanur Rahman: Conceptualization, Methodology, Investigation, Data curation, Formal analysis, Writing-original draft. Anayt Ulla: Methodology, Investigation, Data curation, Formal analysis, Writing-original draft. Honomi Ogura: Data Curation, Formal Analysis. Haruka Tsuda: Data Curation, Formal Analysis. Takayuki Uchida: Investigation, Formal analysis. Tomoya Fukawa: Methodology, formal analysis, visualization. Takeshi Nikawa: Conceptualization, Methodology, Visualization, Funding Acquisition, Project administration, Writing-review and editing, Supervision.

## Funding

This work was supported by Grant-in-Aid for Scientific Research (KAKENHI) (Grant Number: JP23K24770), Japan.

## Declaration of competing interest

All authors have nothing to decalre as the conflict of ineterest.

## Data Availability

The data underlying this article will be shared at reasonable request to the corresponding author.
